# Real-World Data of Esophageal Malignancies Managed Under the Government Scheme From a Tertiary Cancer Center in South India

**DOI:** 10.7759/cureus.77850

**Published:** 2025-01-22

**Authors:** Ravi Teja, Rekabtala Bhaskar, Deepak C Yadlapalli, Sindhusha Kukunuri, Ravi Mohan

**Affiliations:** 1 Medical Oncology, GSL (Ganni Subbalakshmi) Medical College, Rajahmundry, IND

**Keywords:** concurrent chemoradiotherapy, esophageal cancer, india, neoadjuvant treatment, nonmetastatic

## Abstract

Background: Esophageal cancer is a major problem in India. The incidence has a geographic variation, being more common in some parts of south India and pockets in the north. The patients usually present in late stages as the symptoms are non-specific, hence patients are treated for other causes over prolonged periods. There has been marked improvement due to the incorporation of a multi-modality approach in the management of these cancers over the last decade.

Objective: This study included patients with non-metastatic esophageal cancer who presented to the department of medical oncology between January 2020 and December 2023. The aim was to analyze the clinical and demographic profiles and survival outcomes of patients.

Methodology: This is a retrospective study conducted at a tertiary cancer care center in southern India, primarily serving patients from rural areas. Approval from the Institutional Ethics Committee was secured prior to the study (GSLMC/RC:1259A-EC/1259A-05/2024). Case files for all esophageal cancer patients treated at our center between January 2020 and December 2023 were collected from the medical records department and analyzed. We focused on patients diagnosed with non-metastatic esophageal cancer who had received treatment.

Results: A total of 47 patients participated in our study, with a mean age of 55 years. The male-to-female ratio was 2:3. Among the participants, 18 (38.3%) were smokers and 12 (25.5%) were alcohol users. The most frequently affected site was the middle thoracic esophagus, with 22 patients (46.8%), followed by the lower third in 15 patients (31.9%), and both the upper one-third and gastroesophageal junction cancers each accounting for five patients (10.6%). Squamous cell carcinoma was the predominant histological type, representing 82% of cases. Stage 2 was the most common stage at presentation, seen in 22 patients (46.8%), followed by stage 3 in 17 patients (36.2%). Twenty patients received neoadjuvant chemoradiotherapy (NACRT), with 13 (65%) receiving a weekly regimen of paclitaxel and carboplatin, while seven (35%) were treated with a CAPEOX (capecitabine and oxaliplatin) regimen alongside radiation (41.4 to 45 Gray). Of these, only eight patients (40%) proceeded to surgery, while 12 patients (60%) did not. Among those who underwent surgery, five patients (62.5%) achieved a pathological complete response. Additionally, 24 patients received definitive CRT, resulting in a complete response in 14 patients (58.3%) and a partial response in 10 patients (41.6%). Three patients defaulted on treatment. The median overall survival for the analyzed group was 10.5 months, with a subset analysis showing that those who had surgery had a survival duration of 12 months, compared to eight months for those who did not undergo surgery.

Conclusion: The study concluded that the middle thoracic esophagus is the most prevalent site for esophageal cancer, with squamous cell carcinoma being the predominant histological type. Stage 2 is frequently observed at presentation, followed by stage 3. The standard treatment for locally advanced esophageal malignancies now involves a combined approach with NACRT. Despite advancements in multimodal treatments, the prognosis for esophageal cancer remains poor and requires significant improvement.

## Introduction

Esophageal cancer (EC) accounts for the eighth most commonly diagnosed cancer and it is in sixth position worldwide as a leading cause of cancer deaths [1]. Histologically, most ECs are classified into squamous cell carcinomas (SCCs) and adenocarcinomas (ADCAs). When stratified by anatomical location, the incidence of ADCA of the distal esophagus and gastroesophageal junction (GEJ) is increasing rapidly due to Barrett's esophagus [2]. Even for nonmetastatic disease that undergoes potentially curative surgery, outcomes are poor, with five-year survival rates ranging from 20% to 35% [3]. Several approaches have been evaluated to improve these outcomes, including screening for early disease detection, optimal surgical techniques to improve curative resection and reduce perioperative morbidity, and combined treatment protocols.

Global burden

The global incidence of EC shows significant variation, with the highest rates found in the Asian region, particularly in Iran, Turkey, and north-central China [4]. In contrast, Western countries report much lower incidence rates. However, in recent decades, there has been a notable increase in diagnoses of ADCA, largely attributed to rising obesity rates, Barrett's esophagus, and chronic gastroesophageal reflux disease [5]. Countries with a higher Human Development Index (HDI) tend to have increased rates of esophageal ADCA; for instance, the United States has seen a more than 400% rise in esophageal ADCA cases over the past 25 years [6]. Additionally, similar trends have been observed in Asian nations like China and Singapore [7]. The decline in esophageal SCC incidence in certain countries is believed to correlate with economic growth and improved nutrition, while in some high-income nations, this decline is mainly due to reduced smoking rates. Conversely, India exhibits a higher incidence of esophageal SCC, consistent with trends seen in countries with lower HDI.

Burden in India

In India, EC ranked as the sixth most common cancer in 2018, accounting for 4.9% of all cancer cases and 5.9% of cancer-related deaths. That year, there were 52,396 new EC cases and 46,504 deaths attributed to the disease. Among men, EC was among the top five cancers, with 33,890 new cases reported, while it did not appear in the top five cancers for women. Certain regions of India, such as Jammu and Kashmir and most states in northeast India, exhibit particularly high incidences of EC [8].

The poor outcomes associated with surgical resection alone have led to significant research into combined treatment strategies for EC. These strategies include neoadjuvant chemotherapy or chemoradiotherapy prior to surgery, as well as adjuvant chemotherapy, radiotherapy, or chemoradiotherapy following surgical intervention. Most of these approaches have been assessed through randomized trials and meta-analyses. However, there remains a considerable lack of consistency in the treatment protocols for EC [9].

The rationale of neoadjuvant chemotherapy

The potential benefits of neoadjuvant therapy include enhancing the rate of curative resections through tumor downstaging, addressing micro-metastasis earlier, and ensuring more effective delivery of non-surgical treatments due to the tumor's intact vascular supply and the avoidance of extended recovery times following major surgery. The underlying biological rationale involves reduced risk of tumor seeding during surgery and increased radiosensitivity resulting from improved tumor oxygenation when radiotherapy is administered prior to surgery. Additionally, the presence of visible disease facilitates the definition of radiation target volumes. This approach also provides a unique opportunity to assess the effectiveness of preoperative treatment through histological examination of the resected esophagus. Furthermore, patients likely to experience early relapse due to hidden metastatic disease may be spared from surgical resection, effectively selecting patients for treatment based on their prognosis [9].

## Materials and methods

Data of all esophageal cancer patients attending the Department of Medical Oncology, Ganni Subbalakshmi (GSL) Medical College, Rajamahendravaram, Andhra Pradesh, India, were collected from the medical records department between January 2020 and December 2023 and analyzed. Prior to enrollment, all participants were provided with a detailed explanation of the study's aims, procedures, potential risks, and benefits, and written informed consent was obtained. This process was reviewed and approved by the GSL Medical College & Hospital Ethics Committee (GSLMC/RC:1259A-EC/1259A-05/2024).

The study included 47 patients, and the majority were from rural areas. Patients above 18 years of age, with an Eastern Cooperative Oncology Group (ECOG) performance status of 0 to 2, and non-metastatic treatment-naive esophageal carcinoma were included in the study. Patients with an ECOG score of 3 or greater and comorbidities like abnormal renal and liver function, patients who received prior therapy, and patients with metastases or recurrence were excluded.

After discussing in a multidisciplinary tumor board, based on the performance status, age, comorbidities of the patient, and stage, patients were offered different treatment approaches. Patients who were deemed fit for surgery were planned for neoadjuvant chemoradiotherapy (NACRT) administered as follows: paclitaxel (50 mg/m2) and carboplatin (AUC 2) were given intravenously once a week for five weeks, concurrent with radiation therapy delivered at a dose rate of 2 Gy per session, five sessions per week, for a total of 41.4 to 45 Gray followed by trans-hiatal esophagectomy. Patients who were unfit or unwilling for surgery were planned for radical radiotherapy to a dose of 50.4 Gray with capecitabine and oxaliplatin (CAPEOX) after explaining in detail the merits and demerits of each protocol. Patients were followed regularly after treatment. Clinical and demographic profiles, anatomical sites, histology, stage distribution, and survival patterns along different treatment modalities were analyzed. Statistical analysis was performed using SPSS software version 20.0 (IBM Corp., Armonk, NY) and Microsoft Excel 2016 (Microsoft Corporation, Redmond, WA) and was used to plot Kaplan-Meier curves for overall survival.

## Results

A total of 47 patients with non-metastatic EC were included in the study. The mean age at diagnosis was 55 years. The male-to-female ratio was 2:3, with 18 males and 27 females. Among the patients, 18 (38.3%) were smokers, and 12 (25.5%) were alcohol users. Table 1 depicts all the patient characteristics, including age, gender, comorbidities, and habits like smoking or alcohol consumption.

**Table 1:** Patient characteristics (N = 47). Patient specifics including age, sex, history of smoking and alcohol consumption, diabetes mellitus, and hypertension.

	N	%
Age	47	Mean age: 55 years
Sex		
Female	29	61.7
Male	18	38.3
Smoking		
No	29	61.7
Yes	18	38.3
Alcohol		
No	35	74.5
Yes	12	25.5
Diabetes mellitus		
No	43	91.5
Yes	4	8.5
Hypertension		
No	43	91.5
Yes	4	8.5

The disease characteristics are illustrated in Table 2. The most frequent site of presentation for EC is the middle thoracic esophagus, with 22 patients (46.8%), followed by the lower third with 15 patients (31.9%), and both the upper third and GEJ cancers contributing five patients (10.6%) each. SCC is the predominant histological type, accounting for 82% of cases. Stage 2 is the most commonly observed stage at presentation, seen in 22 patients (46.8%), followed by stage 3, which accounts for 36.2% of cases.

**Table 2:** Tumor characteristics (N = 47). GE junction: gastroesophageal junction; upper, middle, and lower 1/3^rd^: upper one-third, middle one-third, and lower one-third of the esophagus. Staging is according to the American Joint Committee on Cancer Tumor, Node, and Metastasis staging, 8th edition.

Site of disease	N	%
GE junction	5	10.6
Lower 1/3^rd^	15	31.9
Middle 1/3^rd^	22	46.8
Upper 1/3^rd^	5	10.6
Histology		
Adenocarcinoma	8	17.0
Squamous cell carcinoma	39	83.0
Stage		
Stage 1	1	2.1
Stage 2	22	46.8
Stage 3	17	36.2
Stage 4	7	14.9

**Figure 1:**
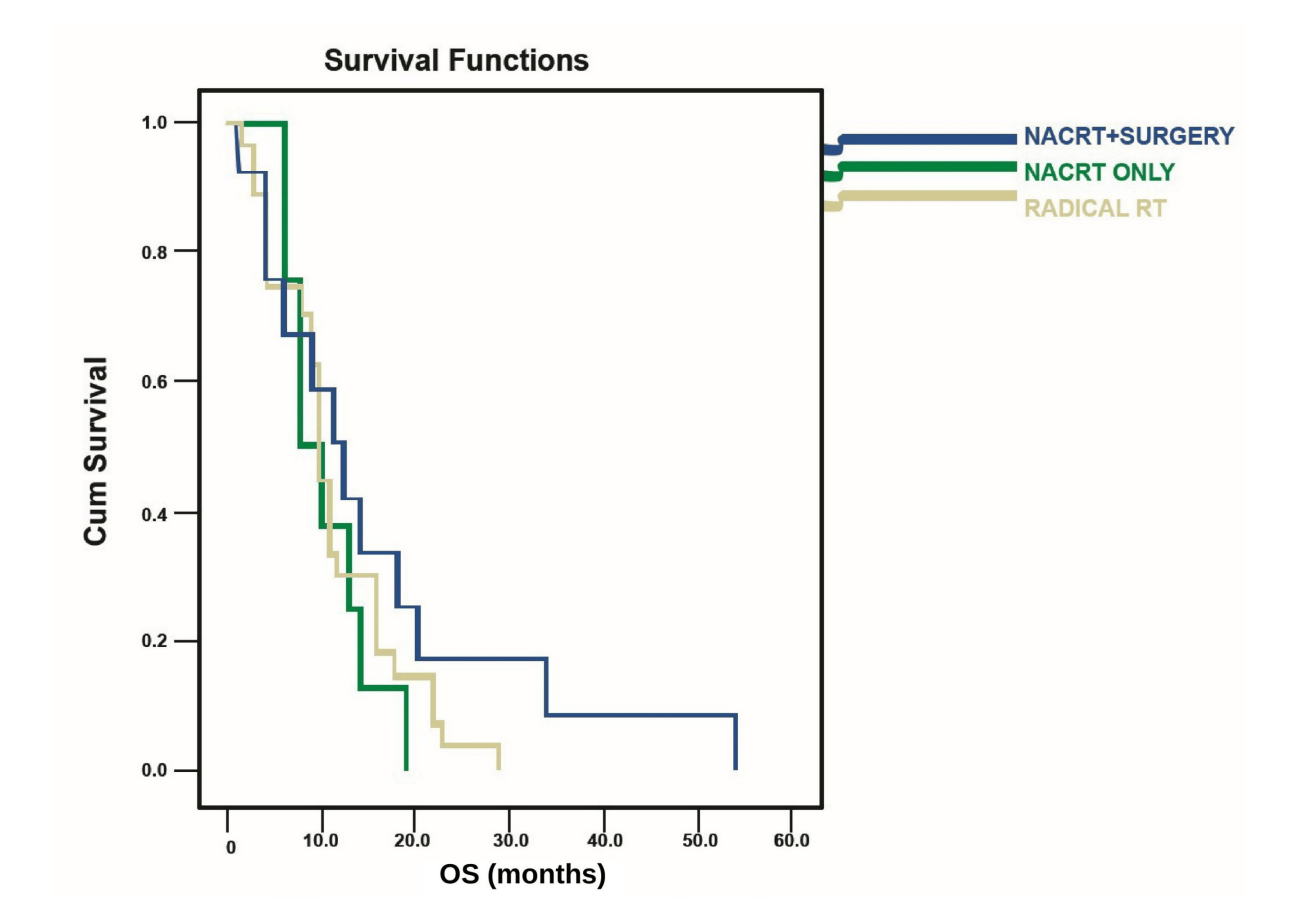
Kaplan-Meier survival curve. OS: overall survival; Cum survival: cumulative survival; NACRT: neoadjuvant chemoradiotherapy; RT: radiotherapy.

A total of 20 patients (42.5%) received NACRT, with 13 of them (65%) treated with a weekly regimen of paclitaxel and carboplatin, while seven patients (35%) received the CAPEOX regimen every two weeks for three cycles, alongside radiation therapy (41.4 to 45 Gray). Among these patients, only eight (40%) proceeded to surgery, whereas 12 (60%) did not. Of those who underwent surgery, five patients (62.5%) achieved a pathological complete response. Additionally, 24 patients (51%) received definitive chemoradiotherapy (CRT), resulting in eight patients (33.3%) achieving a complete response, 10 patients (41.6%) experiencing a partial response, four patients (16.6%) having stable disease, and two patients (8.3%) showing progressive disease. Three patients (6.3%) defaulted on treatment without receiving any intervention. The median overall survival for the analyzed cohort was 10.5 months; in subset analyses, patients who had surgery had a survival duration of 12 months, compared to eight months for those who did not undergo surgery, while patients receiving radical radiotherapy had a median survival of 11.5 months. The survival outcomes are depicted in Figure 1.

## Discussion

The mean age at presentation was 55 years in this single institute-based study. This is similar to the picture in other parts of the country. In a study by Singh et al., the mean age at diagnosis of esophageal malignancy was 58 years [10]. The male-to-female ratio was 1.675, which is similar to the ratio in a study done by Singh et al. [10]. In our study, 18 (38.29 %) patients were habituated to smoking in one or the other form, and 12 (25.5%) were habituated to alcohol. These numbers are much less compared to the studies done in India. In a study conducted by Singh et al. [10], 63% of the patients were smokers and 42% were alcoholics.

The most common site of presentation in our study was the middle one-third (22, 46.8%), followed by the lower one-third (15, 31.9%). SCC was the common histology in 39 (82%) patients in our study, followed by adenocarcinoma in eight (18%) patients. In a study by Singh et al. [10], 96.6% of the patients were of squamous histology and less than 1% were of adenocarcinoma. Stages 3 & 4 (52%) were the most common stage presentation in our study, followed by stages 1 & 2 (9, 48%%).

In our study, based on age, performance status, comorbidities, site of the disease, histology, willingness for the surgery by the patient and their attendants, and nutritional status, patients were considered for NACRT followed by surgery or radical radiotherapy with or without concurrent chemotherapy.

Twenty (42.5%) patients received NACRT, in which 50% of the patients were considered for NACRT and 40% for radical radiotherapy. Eight (40%) patients underwent surgery post NACRT in our study, which is less compared to the other studies within India and the Western world [11]. The main difference in patients undergoing surgery post NACRT might be due to institutional practice in considering borderline fit patients for surgery. Five patients (10.6%) had pathological complete response (pCR) in our study. The pCR rates after surgery for patients who received NACRT range from 15% to 25%. In Western countries, pCR rates typically range from 20% to 30%. In a study done by Mariette et al. [12], pCR rates were 30%.

Twenty-four (51%) patients received definitive CRT. Of these, eight patients (33.3%) achieved a complete response, 10 (41.6%) had a partial response, four (16.6%) had stable disease, and two patients (8.3%) progressed. These response rates indicate the effectiveness of radical radiotherapy as a treatment modality for EC, though outcomes can vary based on patient selection and treatment protocols.

Treatment of EC is a controversial issue and research is focusing on two approaches. The first method involves selecting patients with clinical complete response (cCR) for clinical evaluation, while the second method suggests increasing the radiation dose to treat all patients with the possibility of avoiding surgery. The first approach faces difficulties due to the stringent selection of appropriate patients, but that is the key to assessing the correct response. In contrast, the second approach demands maximized control of local disease. Research results show that only 23% to 40% of cCR patients achieve a pCR post surgery, thus the presence of cCR is not a true reflection [11]. However, reports show that the survival benefit of different anatomic strategies is non-inferior to planned esophagectomy after NACRT [13].

Unfortunately, there are currently no clear guidelines for clinical trial survival. More precisely, Stahl et al. in 2005 concluded that overall survival rates were similar between radical CRT (at least 65 Gy) and surgery (40 Gy) after CRT [14]. The addition of surgical intervention to chemotherapy improves local tumors but does not improve survival outcomes in patients with regional node-involved esophageal SCC. A similar approach was adopted in the FFCD 9102 study, where consolidation supplemented with NACRT (an additional 15-20 Gy), was an alternative to surgery, and no benefit was seen between continuous CRT and surgery [15].

Patients who underwent surgery post NACRT had the highest survival rate among the three groups, followed by the radical radiotherapy group at 11.5 months. In our study, the main causes of noncompliance to surgery after NACRT were considered to be preoperative malnutrition and apprehension toward a morbid surgery.

Our study provides insight into the clinicodemographic profile of EC in south India. Even though all the treatment procedures were covered under the government scheme, the following were the reasons for not undergoing surgery post NACRT in our study: (a) subjective response of symptoms made unwilling for morbid surgery; (b) poor general condition post NACRT; (c) lack of caregiver support post surgery during the hospital stay. These reasons may impact the survival outcomes. These can be overcome by counseling the patient and caregivers regarding the importance of nutrition from day one of admission and explaining the survival advantage of surgery and other adjuvant options, which may increase compliance rates. Most of the patients are of low socioeconomic background and this government scheme impacts their survival and quality of life by providing different modalities of treatment and also providing financial support for rehabilitation after planned treatment.

Our study is retrospective, observational, and noncomparative in nature. The study sample size is too small for any definitive conclusions. We have not used adjuvant immunotherapy in patients with residual disease post surgery, as indicated due to financial constraints under the government scheme.

## Conclusions

Despite advancements in multimodal treatment strategies, the prognosis for EC remains poor and requires improvement. The overall survival rate for EC is low, emphasizing the need for improved treatment outcomes. In our study, the middle thoracic esophagus is the most common site for EC, with SCC being the predominant histological type. Stage 2 is frequently observed at presentation, followed by stage 3. The standard treatment for locally advanced esophageal malignancies now involves a combined approach using NACRT.

Reasons for non-compliance to planned surgery must be addressed, which may increase the rate of planned surgery post NACRT, which will give more insight regarding pathological complete response rates as well as survival rates. Although NACRT has become a standard care approach, ongoing research on adjuvant immunotherapy post surgery needs long-term data validation, which is vital to enhance patient prognosis and address the challenges associated with this disease and its management.
